# Formulation and Evaluation of Supramolecular Food-Grade Piperine HP β CD and TPGS Complex: Dissolution, Physicochemical Characterization, Molecular Docking, In Vitro Antioxidant Activity, and Antimicrobial Assessment

**DOI:** 10.3390/molecules25204716

**Published:** 2020-10-14

**Authors:** Syed Sarim Imam, Sultan Alshehri, Talal Abdullah Alzahrani, Afzal Hussain, Mohammad A. Altamimi

**Affiliations:** Department of Pharmaceutics, College of Pharmacy, King Saud University, Riyadh 11451, Saudi Arabia; sarimimam@gmail.com (S.S.I.); Alzahrani-talal@hotmail.com (T.A.A.); amohammed2@ksu.edu.sa (A.H.); maltamimi@ksu.edu.sa (M.A.A.)

**Keywords:** piperine, HP β CD, food-grade natural compound, antioxidant, molecular docking

## Abstract

The purpose of the present study was to improve the aqueous solubility, dissolution, and antioxidant activity of the water-insoluble drug piperine (PIP). The study was performed by preparing PIP binary inclusion complex (PIP BIC) and piperine ternary inclusion complex (PIP TIC) by different methods. The effect of a hydrophilic auxiliary substance (TPGS) was assessed with addition to PIP and hydroxypropyl beta cyclodextrin (HP β CD) complex. The phase solubility study was performed to evaluate the complexation efficiency and stability constant. The aqueous solubility, dissolution, physicochemical assessment, antioxidant activity, antimicrobial activity, and molecular docking were further evaluated to check the effect of the complexation of PIP. The stability constant (Ks) value was found to be 238 and 461 M^−1^ for the binary and ternary inclusion complex. The dissolution study results showed a marked enhancement of release in comparison to pure drug. XRD and SEM studies revealed the presence of more agglomerated and amorphous structures of PIP, which confirmed the formation of complexes. The results of DPPH radical scavenging and antimicrobial activity showed a significant (*p* < 0.05) enhancement in scavenging activity for PIP TIC (microwave irradiation (MI)). The docking studies have revealed that the binding affinity of TPGS at the PIP-HP β CD complex was −5.2 kcal/mol.

## 1. Introduction

**Figure 1:**
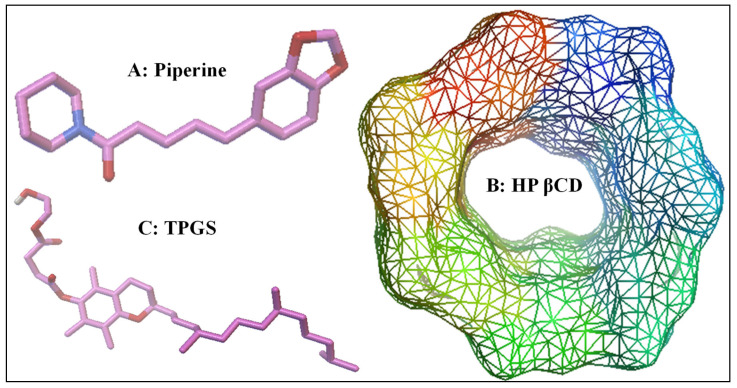
Chemical structure of piperine (**A**), hydroxypropyl β cyclodextrin (HP β CD) (**B**), and d-α-tocopheryl polyethylene glycol succinate (TPGS) (**C**).

Piperine (PIP; Figure 1A) is a pungent alkaloid obtained from black pepper (*Piper nigrum*), long pepper (*Piper longum*), and other *Piper* species (family Piperaceae). The chemical name is (*E*,*E*)-1-piperoylpiperidine and (*E*,*E*)-1-[5-(1,3-benzodioxol-5-yl)-1-oxo-2,4-pentdienyl] piperidine. It is crystalline in nature, having poor water solubility property (40 µg/mL), log P 2.4, and a melting point of 135 °C [1,2]. There are a wide range of pharmacological activities that have been reported for PIP [3,4]. It also has an antimicrobial and antiparasitic effect [4]. Due to its poor solubility, its application as a functional ingredient is limited [5]. 

Cyclodextrins (CDs) are the cyclic oligosaccharides consisting of d-glucopyranose linked by -1,4 glycosidic bonds. There are different types of CDs used to enhance the solubility of poorly water-soluble drugs. The α, β, and γ CDs are classified according to the presence of a number of d-glucopyranose units. CDs have an internal hydrophobic cavity and an outer hydrophilic surface. It forms the inclusion complex with different hydrophobic guest molecules by hydrophobic interaction in aqueous solution [6]. The formed inclusion complex enhances the solubility by several orders of magnitude in comparison to a pure drug. The formation of an inclusion complex with poorly soluble drugs increases drug solubility, dissolution, and bioavailability. It also reduces the gastrointestinal irritancy and masks the bitter taste [7,8]. It can increase the solvation property of the dissolved drug and also stabilize supersaturated solutions [9]. Different types of CDs are widely available and reported to enhance the water solubility of poorly water-soluble drugs. Among these types, hydroxypropyl β CD (HP β CD; Figure 1B) has showed maximum solubility in water (<60% at 25 °C) [10].

Recently, a plethora of research reports published have reported the fact that the solubility and complexation efficiency of CDs can be enhanced by the addition of an auxiliary agent. The addition of an auxiliary/ternary substance into the inclusion complex that interacts with the CD’s outer surface ensures the formation of co-complexes, which may provide a greater stability constant, complexation efficiency, physicochemical properties, and drug transport compared to binary complexes [9]. 

d-α-tocopheryl polyethylene glycol succinate (TPGS; Figure 1C) is a water-soluble derivative of vitamin E. It has an amphiphilic character and self-aggregation property [11]. Due to its chemical stability and better acceptability, it has been widely used as a solubility enhancer. Furthermore, the Food and Drug Administration (FDA) has approved TPGS as a solubility enhancer in different drug delivery systems [12], and it has also been proved to exhibit P-gp inhibitory action and is widely known to increase the bioavailability [13,14]. 

The hypothesis of the present work was designed to enhance the solubility of PIP with the addition of HP β CD and TPGS. The current study was designed to prepare PIP binary and ternary inclusion complexes using solvent evaporation (SE) and microwave irradiation (MI) methods. The prepared complexes were characterized for solubility study, dissolution study, physicochemical characterization, antioxidant activity, antimicrobial activity, and molecular docking. To the best of our knowledge, the effects of TPGS on the PIP–HP β CD complex have not been examined. Therefore, the present study may have greater significance in the future for the formulation of a PIP ternary inclusion complex (PIP TIC) using a HP β CD and TPGS blend.

## 2. Material and Methods

### 2.1. Material

Piperine (PIP) was procured from Beijing Mesochem Technology Co. Pvt. Ltd. (Beijing, China). Hydroxypropyl beta cyclodextrin (HP β CD) was obtained from Sigma, Germany. d-α-tocopherol polyethylene glycol 1000 succinate (TPGS) was a gift from Antares Health Products Inc. All other chemicals used were of analytical grade. Milli Q water was collected from a purification unit and used for the study. 

### 2.2. Formulation Design

The complexes between PIP–HP β CD and PIP–HP β CD–TPGS were prepared using two different methods (solvent evaporation (SE), microwave irradiation (MI)) [15]. The previously sieved (#80 mesh) powder samples were weighed accurately and used for the binary inclusion complex (PIP–HP β CD) and the ternary inclusion complex (PIP–HP β CD–TPGS)**.**

#### 2.2.1. Physical Mixture 

The binary physical mixture (PIP–HP β CD) and ternary physical mixture (PIP–HP β CD–TPGS) were prepared via a geometric mixing of each component. Accurately weighed quantities of samples were triturated and mixed thoroughly in a mortar and pestle (Table 1). The samples were again sieved through #80 mesh and kept in a desiccator for further use. 

#### 2.2.2. Solvent Evaporation Method

PIP binary inclusion complex (PIP BIC) and PIP ternary inclusion complex (PIP TIC) were prepared by the solvent evaporation method. The calculated and weighed amount of PIP was dissolved in ethanol and HP β CD was dissolved in water (ethanol:water, 3:7 *v*/*v*, 10 mL). Slowly, HP β CD solution was added to the PIP phase with continuous stirring. The solvent was evaporated until a dried mass was formed and further kept in a vacuum oven at a temperature of 50 °C for 48 h for the complete removal of solvents [16]. Similarly, PIP ternary inclusion complex was prepared with the addition of TPGS (0.05% *w*/*w*) to the PIP–HP β CD sample. Finally, the prepared samples were dried, milled, and passed through a sieve (#80 mesh) to get fine particles and stored in a desiccator for further use.

#### 2.2.3. Microwave Irradiation Method

Accurately weighed samples of PIP–HP β CD and PIP–HP β CD–TPGS were taken in a beaker (Table 1). The weighed quantity of PIP and HP β CD for the binary inclusion complex was added to ethanol:water (3:7 *v*/*v*, 10 mL) to make a homogenous paste. The prepared sample was kept in a microwave oven (Samsung ME0113M1; Malaysia) for microwave irradiation at 600 W and 50 °C for 4 min [17]. Similarly, PIP–HP β-CD was taken for the ternary inclusion complex and TPGS was added to the PIP–HP β CD complex. The samples were kept in a microwave oven in the same experimental conditions. The irradiated samples were collected and allowed to cool at room temperature. The dried samples were milled and sieved through a #80 mesh to get uniform and fine-sized samples and stored in a desiccator for further use [18]. 

### 2.3. Phase Solubility Study

The phase solubility study was performed for binary and ternary samples as per the procedure reported by Higuchi and Connors [19]. The study was performed in distilled water by adding an excess amount of PIP. An aqueous solution is prepared with HP β CD (0–10 mM) for the binary mixture and with the addition of TPGS (0.05% *w*/*w*) for a ternary sample. The resulting suspensions were shaken on an orbital shaker for 72 h at room temperature (25 ± 2 °C). The samples were collected from the flask, filtered, and then assayed using a UV spectrophotometer at 341 nm following a suitable dilution. Further, the stability constant (*Ks*) was calculated from the *slope* of the phase solubility graph using the below equation [20].

(1) $$Ks=\frac{slope}{S_{0}\left(1-slope\right)}$$

where, *S*_0_ is PIP’s solubility without additives. The complexation efficiency (*CE*) was also calculated using the below formula [20].

(2) $$CE=\frac{slope}{1-slope}$$

### 2.4. Saturation Solubility Studies

The pure, binary, and ternary complexes (PIP, PIP binary physical mixture (BPM), PIP BIC (SE), PIP BIC (MI), PIP TIC (SE), and PIP TIC (MI)) were evaluated for solubility studies. The excess amount of PIP and prepared samples were added to distilled water (25 mL) at room temperature. The samples were mechanically shaken for 72 h at room temperature. The samples were collected, filtered through the membrane filter, and diluted. Finally, the absorbance was measured at 341 nm in triplicate (n = 3).

### 2.5. Drug Content

The drug content was evaluated to check the amount of PIP loaded in the inclusion complex. The weighed quantity of both inclusion complexes (~5 mg of PIP) were taken and dissolved in methanol. The samples were sonicated (1 min) and filtered. The collected samples were diluted further with a methanol water mixture, and PIP contents were evaluated by UV spectrophotometer at 341 nm. 

### 2.6. Dissolution Study

The dissolution study was performed for PIP, PIP-BPM, PIP BIC (SE), PIP BIC (MI), PIP TIC (SE), and PIP TIC (MI) to evaluate the release time profile. The study was performed as per the procedure with the paddle method in the 16th edition of the Japanese Pharmacopoeia. The dissolution medium (water, 900 mL) was used at a temperature of 37 ± 0.5 °C with 50 rpm [5]. The samples containing PIP (~5 mg) were placed into the dissolution medium. The released samples (5 mL) were removed from the flask at fixed time intervals and replenished with the same volume. The sample solutions were filtered through a 0.45 µm membrane filter and diluted with water/methanol in a ratio of 1/1 as appropriate. The PIP contents were measured using a UV spectrophotometer at 341 nm. The release profile was further used to check the release mechanism via plotting of the data into different release kinetic models using PCP Disso V3 software. The data showed the best linear fit, and the highest R^2^ value was selected as the kinetic model [21,22]. 

### 2.7. X-ray Diffraction (XRD)

The diffraction patterns of the solid samples were evaluated by the prepared X-ray diffractometer (Ultima IV diffractometer, Rigaku Inc., Tokyo, Japan). The samples PIP, PIP ternary physical mixture (TPM), PIP TIC (SE), and PIP TIC (MI) were placed in the sample holder and scanned between 3.0° to 60.0° with the angular scanning rate of 0.5°/min. The characteristic peak of each sample was analyzed by collecting the data by monochromatic radiation (Cu Kα1, λ = 1.54 Å). The scanning was performed at an operating voltage of 40 kV and current of 40 mA. The spectra of the prepared PIP ternary inclusion complexes were compared with pure PIP and the changes in peak transformation were observed. 

### 2.8. Scanning Electron Microscopy

The surface morphology of the pure PIP, carriers (HP β CD, TPGS), PIP TPM, PIP TIC (SE), and PIP TIC (MI) was evaluated using the scanning electron microscope (JSM 6360A, JOEL, Tokyo, Japan). The samples were coated with gold and visualized under the microscope to check morphology.

### 2.9. Fourier-Transform Infrared Spectroscopy

The samples were evaluated to check the complex formation by evaluating the change in peak shape, peak position, and intensity. The spectra of each sample (PIP, β-CD, TPGS, PIP TPM, PIP TIC (SE), and PIP TIC (MI) were compared with each other and the results observed were interpreted. The study was performed using a spectrophotometer (ATR-FTIR, Bruker Alpha, Germany). The samples were analyzed between 4000 and 400 cm^−1^, and spectra were interpreted for any conformational changes. 

### 2.10. Nuclear Magnetic Resonance

An NMR study was performed to evaluate the physical properties and conformational changes in the prepared complex. The study was performed for PIP, PIP TPM, PIP TIC (SE), and PIP TIC (MI), and the spectra were compared. The samples were assessed using ^1^H NMR (700 MHz) and ^13^C NMR (125.6 MHz; Bruker NMR; software Top Spin 3.2, NMR 700 MHz, Bruker, Switzerland) studies. The study was performed using deuterated DMSO using TMS as an internal standard. 

### 2.11. Antioxidant Activity

The antioxidant activity of the pure PIP and PIP TIC (MI) was examined as per the reported procedure with some modifications [23]. Stock solutions (10 mg/mL) of pure PIP and PIP TIC (MI) were prepared in ethanol. The samples were diluted further with ethanol at concentration range of 20–300 µg/mL. From each sample, 500 µL was collected and mixed with freshly prepared 0.02% DPPH in 99.5% ethanol (125 µL). The mixture was shaken vigorously and kept in the dark at room temperature for 60 min. A chemical reaction takes place between the violet colored DPPH solution and antioxidant molecule. After the reaction, the violet colored solution turns into a colorless solution. The presence of electron scavenging capacity of antioxidant molecules helps to change in color of samples. The samples were evaluated at 517 nm using a UV spectrophotometer. Similarly, the blank sample was prepared without addition of PIP, and the antioxidant activity was calculated in triplicate using the equation:

(3) $$Radical\;Scavenging\;\left(\%\right)=\frac{\left(A_{C}-A_{t}\right)}{A_{C}}\;\times \;100$$

where, *A_C_* = control sample absorbance, *A_t_* = test sample absorbance.

### 2.12. Antimicrobial Study

The selected PIP ternary inclusion complex was evaluated for antimicrobial activity using the well diffusion method. The bacterial strains were sub-cultured in a nutrient broth (NB) at specified incubation conditions (following the instruction leaflet). Each strain was exponentially grown to optical density (OD600) of 0.6 (absorbance at 600 nm) and serially diluted to 10^−4^. In order to assess the zone of inhibition (ZOI), a strain (1 mL) with a suitable bacterial load (Table 2) and nutrient agar media previously cooled to room temperature (25 mL) were completely mixed in a sterilized beaker and poured into a Petri dish aseptically. The plates were allowed to solidify, and then wells were created using a sterilized steel borer (6 mm in diameter). The test sample (100 µL) was transferred to the respective well and incubated for 24 h at 37 ± 1 °C. The study was conducted in triplicate against each strain to get the ZOI (mean ± SD). A plate containing growth media only served as control, which ensured the sterility of the growth media and process adopted. 

### 2.13. Molecular Modeling Studies

The molecular modeling studies of PIP with HP β CD in the presence of TPGS were carried out in the ternary inclusion complex by using AutoDock 4.2. Using the Chem 3D 14.0 suite, PIP and TPGS ligand structures were drawn and geometry optimization was carried out using the OPLS2005 force field. The crystal structure of the HP β CD crystal was retrieved and extracted from the PDB co-crystal of β-amylase [(Protein data bank: (PDB) code: 1BFN, resolution, 2.07 Å)]. HP β CD was finally drawn manually by attaching an isopropyl group on the 6-OH group of glucopyranose. Auto Dock Tools (ADT) version 1.5.6 (www.autodock.scrips.edu; La Jolla, CA, USA) was used to prepare the binary inclusion complex of PIP at the HP β CD cavity. Finally, the ternary inclusion complex was generated by docking the binary inclusion complex with TPGS.

### 2.14. Statistical Analysis

The experimental results were analyzed statistically using one-way ANOVA followed by Dunnett test using Graph Pad instant software (San Diego, CA, USA). *p* < 0.05 was considered as a statistically significant value.

## 3. Results and Discussion

### 3.1. Phase Solubility Studies

**Figure 2:**
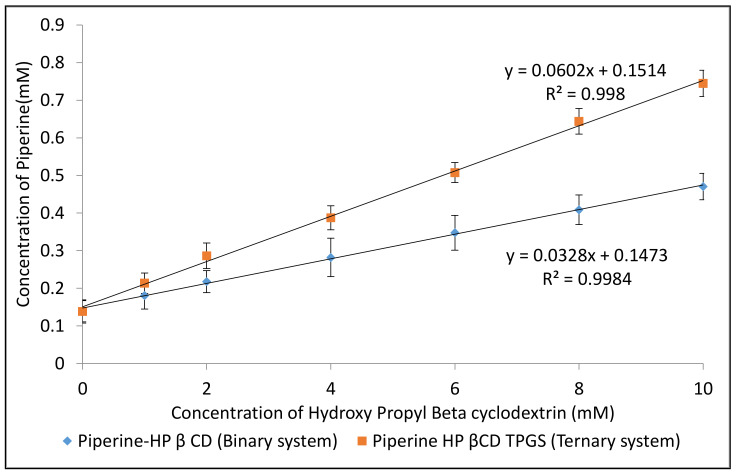
Phase solubility study profile of binary (PIP–HP β CD) and ternary (PIP–HP β CD–TPGS) inclusion complex systems. Values are presented as means ± SD with triplicates.

The phase solubility studies were performed to evaluate the affinity between drug and HP-β CD at 25 °C. The phase solubility graph of PIP with HP β CD in the presence and absence of TPGS (0.05%) was found to be of the A_L_ type as depicted in Figure 2. It indicates a linear increase in drug solubility as a function of HP β CD concentration, leading to the formation of a first-order complex with respect to HP β CD. The slope value for both the complexes was found to be closer to unity. The graph suggested the formation of a 1:1 stoichiometric complex between PIP and HP β CD [24]. The stability constant was calculated from the slope value, and the binary and ternary complexes showed the values of 238 and 461 M^−1^, respectively. The ternary complex showed a significant higher stability constant value, and the value confirmed that the complex was stable [25]. The addition of a ternary substance interacts with the outer surface of the CD as well as the drug–CD complex and helps to form co-complexes or aggregates [26]. It promotes stability constant (Kc) values and also increases the complexation efficiency in comparison to binary complexes. Complexes with a stability constant value between 100 and 1000 mol L^−1^ have biological application. Values lesser than 100 mol L^−1^ give unstable drug–CD complexes, and values higher than 1000 mol L^−1^ adversely affect drug absorption [27]. The significant enhancement in the stability constant and complexation efficiency were achieved with the addition of TPGS as a ternary substance. 

### 3.2. Saturation Solubility Studies

**Figure 3:**
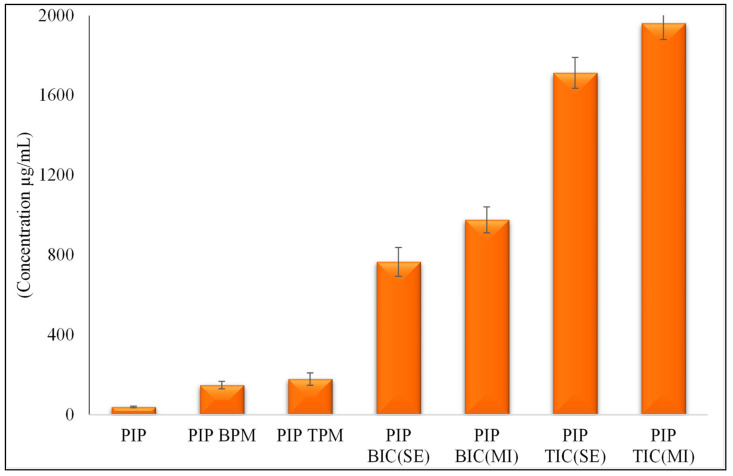
Comparative saturation solubility study of pure PIP and PIP binary and ternary inclusion complex. Values are presented as means ± SD with triplicates.

The reported solubility of pure PIP was found to be 40 µg/mL in water [1], which proved to be in excellent agreement with our study 38.78 ± 4.21 µg/mL (Figure 3). The solubility of PIP in the binary and ternary samples ((PIP BIC (SE), PIP BIC (MI), PIP TPM, PIP TIC (SE), and PIP TIC (MI)) significantly increased compared to that of pure PIP. The binary and ternary physical mixtures (PIP BPM and PIP TPM) depicted a 3.8- and 4.59-fold enhancement in aqueous solubility. The binary inclusion complexes (PIP BIC (SE) and PIP BIC (MI)) showed 20.11- and 25.13-fold enhancement in solubility. The addition of the auxiliary substance TPGS with HP β-CD in the ternary inclusion complex (PIP TIC (SE) and PIP TIC (MI)) produced a synergistic effect in increasing the solubility as compare to that of the binary inclusion complex. The ternary complex (PIP BIC (SE) and PIP BIC (MI)) showed 44.25- and 52.67-fold enhancement in water solubility. The addition of a ternary substance in the drug–CD complex induces interaction with the hydrophobic portion of HP β CD [8]. The direction of the hydrophilic part of TPGS toward the drug and CD decreases the surface tension and leads to enhanced aqueous solubility.

### 3.3. Drug Content

The drug content analysis was performed in triplicate, and the average was reported. The drug contents of the binary inclusion complex (PIP BIC (SE) and PIP BIC (MI)) and ternary inclusion complex (PIP TIC (SE) and PIP TIC (MI)) were found in the range of 98.12 ± 4.56% to 99.81 ± 3.38%, respectively. The results indicated that the drug was uniformly distributed in all the complexes.

### 3.4. Dissolution Study

**Figure 4:**
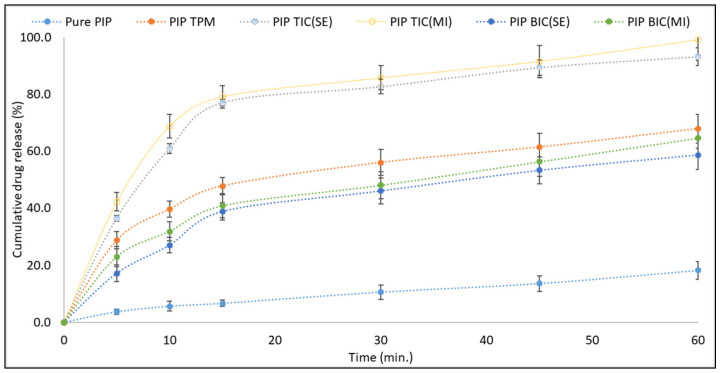
Comparative drug release profile of pure piperine and piperine inclusion complexes (binary and ternary). Values are presented as means ± SD with triplicates.

The release study was performed to check the release pattern of the prepared samples. There was a significant improvement in the release observed for the tested PIP ternary inclusion complex. The order of release was as follows PIP TIC (MI) > PIP TIC (SE)> PIP TPM > PIP BIC (MI) > PIP BIC (SE) > PIP (Figure 4). Pure PIP showed a poor drug release profile of 18.21 ± 3.12% in 60 min. PIP BIC (SE and MI) showed higher drug release than pure PIP. The enhancement in the release was achieved due to an improvement in the solubility of pure PIP by partial entrapment into the HP β CD [22]. PIP TIC (SE and MI) showed significantly higher (*p* ˂ 0.05) drug release than PIP TPM. PIP TIC (SE) and PIP TIC(MI) showed 93.24 ± 3.65% and 99.23 ± 4.11% PIP release compared to PIP TPM (68.43 ± 3.12%). The greater enhancement in the result was found due to the addition of TPGS as a ternary substance. Its presence in inclusion complex may help to get greater inclusion, complexation, and amorphization. TPGS is an amphiphilic molecule, and the lipophilic portion gets attracted toward the CD cavity and may pose competition to the drug. 

The presence of two solubilizers could have shown a synergistic effect on the drug solubilization. Both CD and TPGS could have been available to the drug as solubilizers by inclusion complexation and by surfactant (micellar solubilization) effect, respectively [28]. The concentration of TPGS used to formulate the ternary inclusion complex was 0.05% [9]. There was a slight difference in the release pattern observed between the complex prepared by the MI method and that prepared by the SE method. used PIP TIC (MI) showed greater release than PIP TIC (SE). The application of microwave gives the production of uniform heat to the sample at the same rate and gives better intimate contact between PIP, TPGS, and HP-*β* CD [6,29].

The prepared inclusion complex was also assessed for T_50%_ (time taken to release 50% of PIP) and T_90%_ (time taken to release 90% of PIP). The results showed that PIP TIC (MI) and PIP TIC (SE) had T_50%_ values of 5.3 and 7.5 min, respectively. PIP BIC (MI) and PIP BIC (SE) showed T_50%_ values of 34.2 and 38.8 min, respectively. The T_90%_ values of PIP TIC (MI) and PIP TIC (SE) occurred at 32.2 and 38.3 min, respectively. The release data of PIP TIC (MI) was fitted to different release kinetics model, and the correlation coefficient (R^2^) values were found to be maximum for first-order kinetics. 

### 3.5. XRD Activity

**Figure 5:**
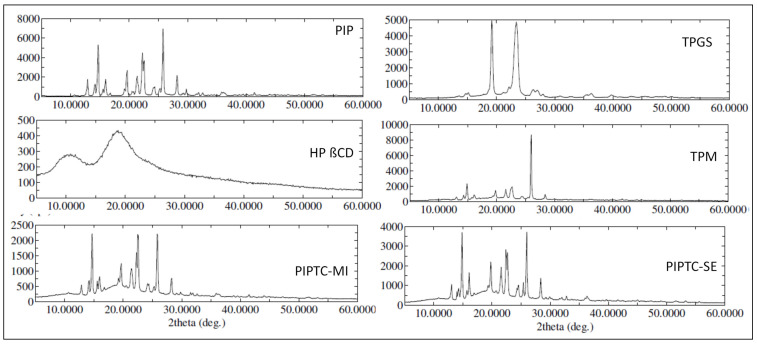
X-ray spectra of pure piperine, TPGS, HP *β* CD, PIP ternary physical mixture, piperine ternary inclusion complex (MI), and piperine ternary inclusion complex (SE).

An XRD study was performed to check the changes in the crystallinity of the pure sample PIP. The comparison was performed between the characteristic peaks of PIP, carriers (HP β CD and TPGS), and prepared samples (PIP TPM, PIP TIC (MI), and PIP TIC (SE)). Results are depicted in Figure 5. PIP showed the sharp intense characteristic diffraction pattern angle between 10° and 30°. It confirms the crystalline nature of the PIP. HP β CD and TPGS showed their characteristic peaks that match with the reference. PIP TPM showed low-intensity peaks, which confirms the partial loss of crystallinity. However, the prepared ternary inclusion complex (PIP TIC (MI) and PIP TIC (SE)) showed significant changes in the PIP characteristic peaks. The high-intensity peaks were reduced, transformed into low-intensity ones, demonstrating the nature of the inclusion compound in compare to that of the pure PIP. The comparisons in the peaks were observed between samples prepared by MI method and SE method. The sample prepared with MI method showed significant low-intensity peaks. The changes observed may be due to the complexation of PIP in HP β CD in the presence of TPGS. 

### 3.6. Scanning Electron Microscopy

**Figure 6:**
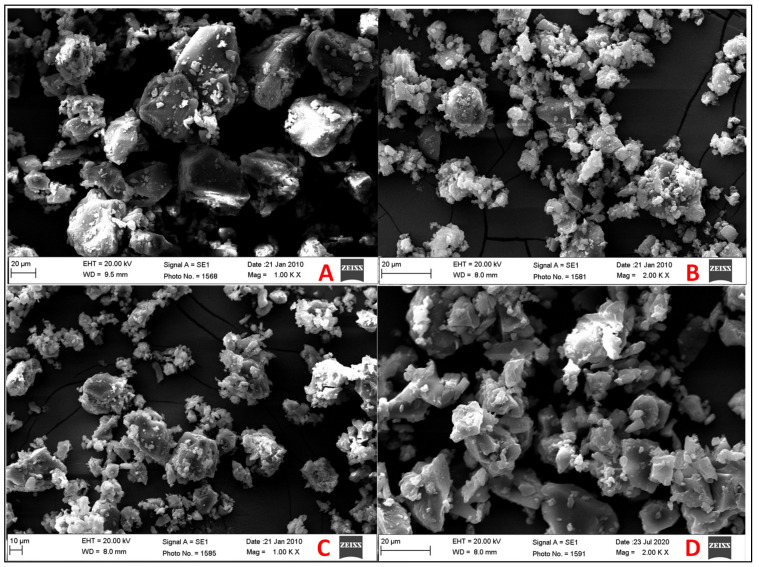
SEM of (**A**) pure piperine, (**B**) PIP ternary physical mixture (PIP TPM), (**C**) PIP ternary inclusion complex ((PIP TIC (MI)), (**D**) PIP ternary inclusion complex ((PIP TIC (SE)).

The surface morphology of pure PIP and the prepared samples was evaluated to check the changes in the particles’ morphology, shown in Figure 6. Pure PIP showed a well-defined crystal shaped structure. The image of PIP TPM showed a lesser amount of PIP crystals with the presence of HP β CD and TPGS. The size of the particles was reduced due to the milling process, but some agglomerates were still present. In the case of PIP TIC (MI) and PIP TIC (SE) images, there is greater transformation from a crystalline state to amorphous agglomerates with uneven and porous surfaces. The change in particle shape and aspect in the inclusion complex prepared by SE and MI methods indicates the formation of a new solid phase as consequence of the crystalline habitus change [30]. The polymorphic transformation and the formation of homogenous agglomerates were due to the close contact between HP β CD and TPGS. These factors may lead to increased solubility and dissolution of drug. The amorphous material dissolves at a faster rate due to its greater internal energy and molecular motion as compared to the crystalline structure [20]. 

### 3.7. Fourier-Transformed Infrared Spectroscopy

**Figure 7:**
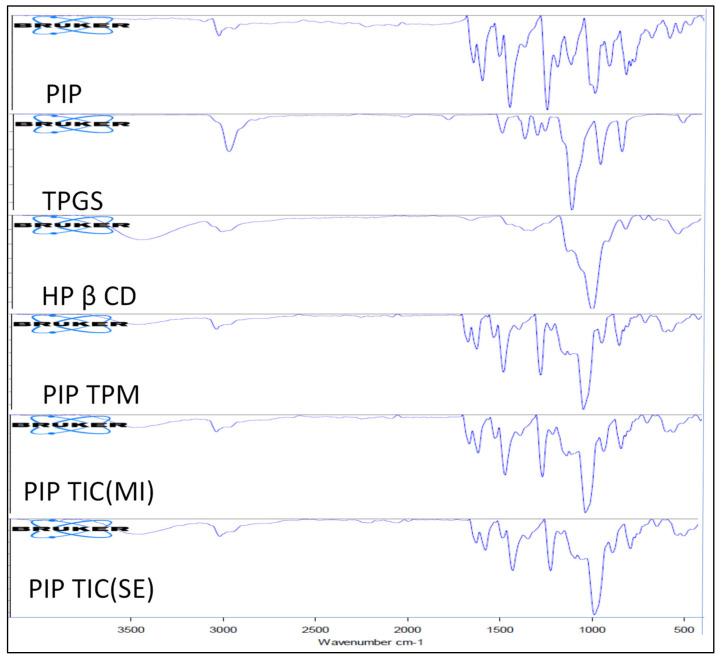
IR spectra of pure piperine, HP β CD, TPGS, PIP ternary physical mixture, piperine ternary inclusion complex (MI), and piperine ternary inclusion complex (SE).

The attributed IR vibrations of the pure drug PIP, carriers HP-β CD and TPGS, and developed formulations ((PIP TPM, PIP TIC (MI), and PIP TIC (SE)) with the functional groups accountable for being congruous with stretching frequencies are represented in Figure 7. PIP, an alkaloid, belonging to the family of nitrogenous compounds, exhibited a sharp stretching spectral peak for tertiary nitrogen at 2931.00 cm^−1^. The characteristic carbonyl stretching vibrations were observed at 1623.22 cm^−1^. The C-O-C, C=C ethylenic and C=C aromatic stretching peaks were also observed at 1121.34, 1577.79, and 1436.28 cm^−1^ for the pure drug, respectively. The featured frequencies for HP β CD were recognized at 3332.62 and 1020.70 cm^−1^, which contemplate the stretching vibration of O–H and C-O-C moiety. The most perceptible peaks for TPGS were the stretching vibration at 2876.80 cm^−1^ for the ester group and 1344.51 cm^−1^ for the C=C aromatic stretching. The carrier also showed a distinct peak for C-O-C stretching at 1100.88 cm^−1^.

The ternary physical mixture (PIP TPM) and ternary inclusion complexes ((PIP TIC (SE) and PIP TIC (MI)) showed the presence of a tertiary nitrogen peak (CH_2_-N), which was also confirmed by ^1^H NMR, as an observed with pure PIP. There was a slight variation in the peaks for C=C aromatic and C-O-C stretching, which corresponded to 1438.87 and 1024.82 cm^−1^, respectively, which might be due to the presence of carriers. Further, C=C ethylenic stretching vibrations were observed at 1581.06 cm^−1^ for TPM and 1577.61 cm^−1^ for TIC (SE). In contrast to pure drug, TIC (MI) showed an absence of the C=C ethylenic stretching vibration peak, which was also further confirmed by NMR spectroscopy. The peak for carbonyl moiety (C=O) of the pure drug PIP was also observed at 1638.21 cm^−1^ in the complexes. The complexes also showed a broad peak of hydroxyl moiety at 3311.52 cm^−1^ of the HP β CD carrier, which may be concluded for the formation of complexes. These findings established that PIP TIC (MI) exhibited greater solubility in the presence of carriers, which was further confirmed by NMR as compared to PIP TPM and PIP TIC (SE).

### 3.8. Nuclear Magnetic Resonance

**Figure 8:**
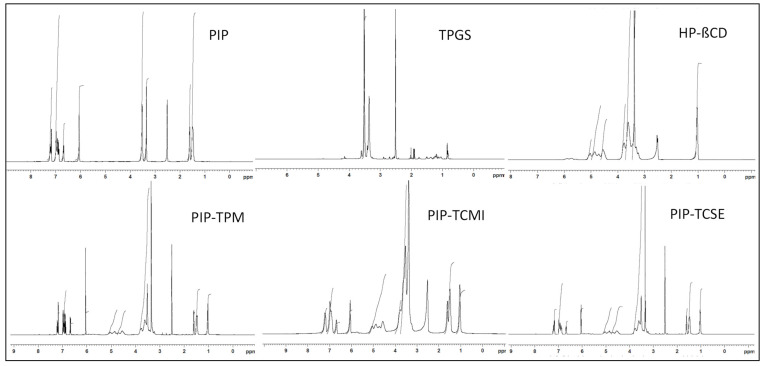
^1^H NMR spectra of pure piperine, HP *β* CD, TPGS, PIP ternary physical mixture, piperine ternary inclusion complex (microwave irradiation), and piperine ternary inclusion complex (solvent evaporation).

**Figure 9:**
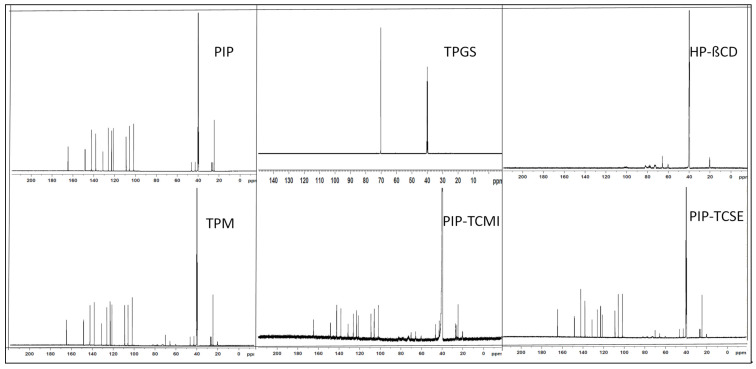
^13^C NMR spectra of pure piperine, HP *β* CD, TPGS, PIP ternary physical mixture, piperine ternary inclusion complex (microwave irradiation), and piperine ternary inclusion complex (solvent evaporation).

The proton and carbon-13 NMRs of pure PIP, PIP TPM, PIP TIC (MI), and PIP TIC (SE) were performed to explore the relations connecting pure PIP and carriers (HP β CD and TPGS^®^) utilizing the chemical shift (δ), as shown in Figure 8 and Figure 9. The chemical shift values of proton NMR spectra of pure PIP, HP β CD, and TPGS were collated with the formed complexes of PIP TPM, PIP TIC (MI), and PIP TIC (SE). The proton NMR spectrum of PIP in DMSO-*d6* showed a distinct singlet at 6.05 ppm, which was attributed to the methylenic proton of benzodioxol. A multiplet peak was observed for the piperidine ring at C-3, C-4, and C-5 having a δ value of 1.50–1.61 ppm. The singlet CH_2_-N peak for the piperidine ring was also observed at δ 3.52 ppm. The structure also exhibited a multiplet peak of benzene at a δ value of 6.87–6.99 ppm. The two distinct peaks for ethylene at position 2’ and 4’ having a sharp singlet peak were also seen at δ 6.67 and 7.22 ppm. The singlet peaks at 3’ and 5’ of ethylenic bond were observed at δ 7.24 and 6.69 ppm. The singlet peak for the HP β CD carrier was explored at δ 5.03 ppm at position 1 of the glucose moiety. The other peaks of glucose moiety were also observed. The singlet peak of the hydroxyl moiety of the carrier was seen at δ 2.51 ppm. The chemical shift values of the second carrier, TPGS, showed a singlet methylene (-CH_2_) peak at δ 3.52 and 3.36 ppm corresponding to the position C-34 and C-35 of the structure. 

In disparity, the samples PIP-TPM, PIP TIC(MI), and PIP TIC(SE), depicted totally imperceptible changes in the δ values of methylene moiety of benzodioxol with a singlet δ value of 6.06 ppm. A minor change was observed for the multiplet peaks of the piperidine ring at C-3, C-4, and C-5 having a δ value of 1.47–1.61 ppm for the formed samples PIP TPM, PIP TIC (MI), and PIP TIC (SE). The singlet CH_2_-N peak of the piperidine ring was also observed at δ 3.51 ppm for the complexes, in compliance with IR spectral values. The samples PIP TPM and PIP TIC (SE) almost exhibited a similar multiplet peak of benzene at a δ value of 6.87–6.99 ppm, but the complex PIP TIC (MI) showed a drastic change in the multiplet peak of the benzene having δ values at 6.93–6.99 ppm, which may be attributed due to the influence of carrier peaks in the inclusion complex. The two distinct singlet peaks for ethylene at position 2′ and 4′ were seen at δ 6.67 and 7.17 ppm and at position 3’ and 5’ were observed at δ 7.23 and 6.70 ppm for the complexes PIP TPM and PIP TIC (SE). In contrast, as already stated in IR spectroscopy, the complex PIP TIC (MI) does not depict an ethylenic bond, instead a multiplet peak of ethane was observed at a δ value ranging from 6.70 to 7.22 ppm. The carrier peaks of HP β CD glucose at δ 5.05–3.35 ppm were also observed in all the complexes with minor changes in chemical shift values, which may indicate the formation of complexes. All the complexes exhibited the value of δ 1.03 ppm for the methyl group of hydroxypropyl at position 9 of the glucose moiety. The peaks of the carrier TPGS of methylene moiety at C-34 were also observed in all the complexes, but C-35 peaks were only observed in the complex PIP TPM at δ 3.43 ppm. The results of proton NMR and IR spectral values of PIP TIC(MI) showed that the HP β CD and TPGS^®^ peaks encountered massive adjustment, which exhibited its gravity in the solubility augmentation of PIP. The hydroxyl moiety of β-CD also exhibited a singlet peak at δ 2.52 ppm with a slight deviation and is also consistent with the IR spectral values. The appearance of the carbonyl group of pure PIP and the hydroxyl group of HP β CD in complexes with minute chemical shift also stipulate the formation of complexes. A significant upfield shift in the presence of HP β CD was observed for aromatic protons of PIP. The presence of a hydroxyl group in complexes as confirmed by the spectroscopy indicates that the carbonyl group from PIP and oxygen atom of succinic ester group of TPGS facing toward the OH group of glucopyranose form a strong hydrogen bond indicating the formation of the complexes, which leads to enhanced PIP solubility.

In ^13^C NMR, the complexes exhibited a slight variation in the peaks for carbonyl value at δ 164.72 ppm as compared to the pure PIP having δ value of 164.69 ppm. Extra peaks were observed for the complexes, which may be attributed to the carriers HP β CD or TPGS. The chromenyl moiety of TPGS at δ 72.81 ppm was missing in all the prepared samples. The C-13 values also showed the presence of a piperidine ring in all the complexes at δ value of 46.55 ppm at C-2,6, δ 26.96 ppm at C-5, δ 25.89 ppm at C-3, and δ 24.63 ppm at C-4. The result was consistent with the proton NMR and IR spectroscopy. The presence of glucose and succinate peaks of the carrier HP β CD and TPGS at δ 102.11 ppm and δ 70.25 ppm in all the complexes further confirmed the formation of complexes. The above spectroscopic values also confirmed that there is no interaction between the pure drug, carriers, and the formed complexes. The IR and NMR spectral values also confirmed that the complex PIP TIC (MI) showed a significant solubility enhancement in presence of the carriers. The above statement is concluded on the basis of the explanation given by the various spectral characteristic values, which is further supported by molecular docking studies.

### 3.9. Antioxidant Activity

**Figure 10:**
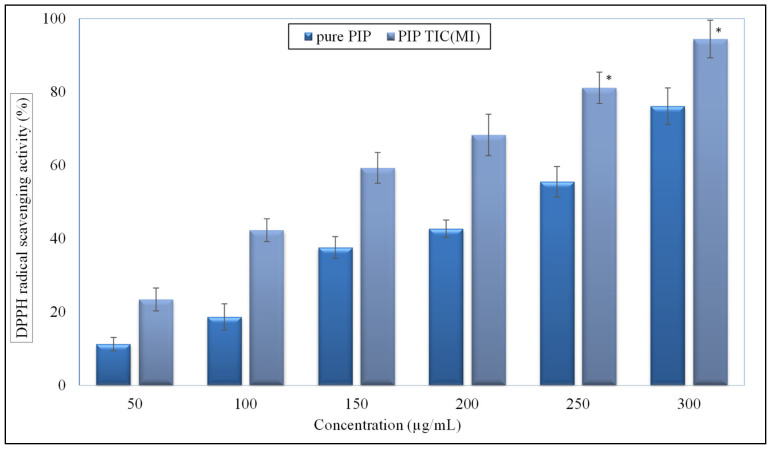
Comparative antioxidant activity profile of pure piperine and piperine ternary inclusion complex (PIP TIC (MI)). Values are presented as means ± SD with triplicates. The values having superscript (*) were significantly different (*p* < 0.05).

DPPH is a stable free radical substrate used to assess the antioxidant activity [31]. It can react with a proton donor groups and change color to violet. The comparative antioxidant activity results between pure PIP and PIP TIC (MI) are depicted in Figure 10. As can be deduced from the figure, the scavenging activity of the tested samples was directly dependent upon the concentration. The result indicates that there was a significant enhancement in the antioxidant activity observed for both PIP and PIP TIC (MI) as their concentration increased. PIP TIC (MI) showed a maximum activity of 94.43% and pure PIP showed 76.12% at the same concentration (300 µg/mL). The difference was found significant (*p* < 0.05) in the activity vis a vis pure PIP. The higher activity achieved by the PIP TIC (MI) was due to the greater solubility of PIP in the presence of HP β CD and TPGS. The antioxidant donates hydrogen or electron to DPPH to convert it to DPPH-H [32]. 

### 3.10. Antimicrobial Activity

The antimicrobial activity of the prepared samples was evaluated against different microorganisms, and the results were compared with pure PIP. The ZOI against four strains is shown in Table 2. Pure PIP showed a ZOI of 12.4 ± 0.48, 8.8 ± 0.78, 11.3 ± 0.69, and 7.2 ± 0.56 mM against *Staphylococcus aureus*, *Bacillus subtilis*, *Escherichia coli*, and *Enterococcus faecalis*, respectively. The prepared samples PIP TPM and PIP TIC (MI) showed enhanced antibacterial activity against all the tested organisms due to the marked enhancement in solubility. The sample PIP TIC (MI) showed greater ZOI than pure PIP and PIP TPM. An enhanced ZOI result was found for PIP TIC (MI) as 15.7 ± 0.73, 13.8 ± 0.46, 16.4 ± 0.36, and 11.5 ± 0.53 mm against *S. aureus, B. subtilis, E. coli,* and *E. faecalis*, respectively. It is apparently obvious that the complex is more significantly (*p* ˂ 0.05) sensitive against *B. subtilis* and *E. coli* as evidenced by the values of ZOI (Table 2). The above findings may be due to the higher solubility of PIP inclusion complex and maximized internalization within the bacterial cell, leading to breakdown of the cytoplasmic membrane (oozing of cytoplasmic content and cell wall fragmentation) and the cell wall [33,34]. In addition, PIP is a potent antibacterial agent acting as an inhibitor of the efflux pump of *S. aureus* resulting in accumulation of PIP to a toxic level in the bacterial cell membrane [35]. This finding of the study is of great importance for the tested organisms. There are several reports on the challenges faced by the scientists due to frequent development of resistance toward antibiotics and various enterotoxins causing several types of enteritis and septicemia [31]. Thus, the improved antibacterial activity of PIP may be attributed to enhanced solubility of the drug using US-FDA-recommended safe and GRAS (generally regarded as safe)-grade (food-grade) cyclodextrin. This finding can be implemented in formulation design intended for oral, topical, and parenteral drug delivery at an economic scale.

### 3.11. Molecular Docking

**Figure 11:**
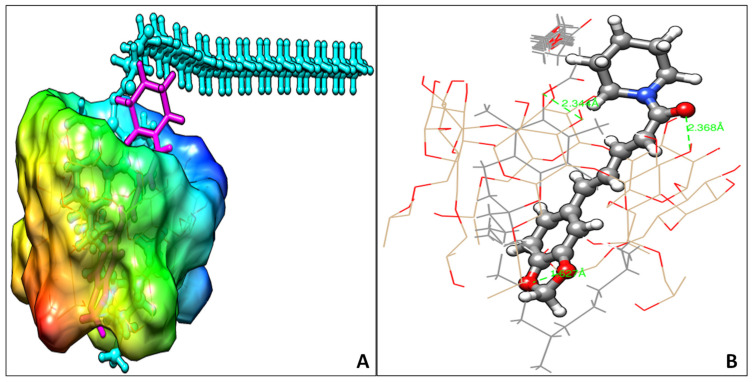
(**A**) The side binding poses of the ternary inclusion complex piperine (magenta), HP β CD (surface view), and TPGS (cyan), (**B**) binding mode of PIP (ball and stick), TPGS (stick) at the central cavity of HP β CD (wire). Piperine formed two hydrogen bonds (green dotted lines) with 3-OH (2.36 Å) and hydroxy propyl (1.62 Å), whereas TPGS formed one hydrogen bond with 3-OH (2.34 Å).

In our effort to understand the stable molecular arrangement in the inclusion complexes, we focused on the molecular interactions of PIP and HP β CD with TPGS. Auto Dock 4.2 with a Lamarkian genetic algorithm-implemented program suite was employed to identify appropriate binding modes and conformation of the ligand molecules. The docked conformations of the two ligands PIP and TPGS bound to the HP β CD are shown in Figure 11A,B. The docking studies have revealed that the binding affinity of PIP at the HP β CD was −7.2 kcal/mol. The binding interaction of binary complex revealed that PIP was aligned in the central cavity of HP β CD by forming two hydrogen bonds. The benzodioxolyl ring occupied the HP β CD cavity, whereas the terminal piperidine ring protruded out of the cavity. The carbonyl group from PIP faced toward the 3-OH group of glucopyranose of HP β CD and formed a strong hydrogen bond with a bond length of 2.36 Å. The oxygen atom of the benzodioxolyl ring formed a hydrogen bond with the OH group of the propyl side chain of HP β CD (1.62 Å). The aromatic benzodioxolyl ring of PIP occupied the hydrophobic portion inside the HP β CD and formed several CH–π interactions with the propyl side chain. The supramolecular ternary inclusion complex of PIP, HP β CD, and TPGS was modeled by docking TPGS on the binary inclusion complex of PIP and HP β CD (Figure 11A). The docking studies have revealed that the binding affinity of TPGS at the PIP–HP β CD complex was −5.2 kcal/mol. The molecular interaction studies of TPGS showed that the chromenyl moiety along with the aliphatic side chain occupied the central cavity of HP β CD whereas, the succinate and polyethylene glycol chain remained outside HP β CD. The 3-OH group of the glucopyranose unit formed one hydrogen bond with an oxygen atom of the succinic ester group of TPGS (2.34 Å). The aliphatic side chain of TPGS occupied the hydrophobic pocket of HP β CD by the hydrophobic interactions. No steric hindrance has been observed between the PIP and TPGS molecules when put together inside HP β CD cavity (Figure 11B), hence the system might be considered as a thermodynamically stable ternary inclusion complex.

## 4. Conclusions

The present study was designed to prepare and evaluate the piperine ternary inclusion complexes by microwave and solvent evaporation methods. The binary and ternary systems showed stability constant (Ks) values of 238 and 461 M^−1^, which confirm that the prepared complexes were stable. The drug content was found also satisfactory. A significant enhancement in the solubility and dissolution was achieved with the prepared inclusion complex. XRD and SEM study revealed the presence of more agglomerated and amorphous structures of PIP. The spectroscopic (IR and NMR) results also confirmed the formation of inclusion complex between the pure drug and carriers. The result of DPPH radical scavenging activity showed a significant (*p* < 0.05) enhancement in scavenging activity for PIP TIC (MI). Due to the marked enhancement in the solubility, the antimicrobial activity of the prepared samples PIP TIC (MI) showed enhanced antibacterial activity against all the tested organisms. The above statement is concluded on the basis of the explanation given by the various spectral characteristic values, which is further supported by molecular docking studies.

## Figures and Tables

**Table 1:** Formulation design of piperine inclusion complex using different methods (*w*/*w*).

Piperine Binary Complex			Piperine Ternary Complex		
Physical Mixture	Solvent Evaporation	Microwave Irradiation	Physical Mixture	Solvent Evaporation	Microwave Irradiation
Piperine:HP β CD	Piperine:HP β CD	Piperine:HP β CD	Piperine:HP β CD: TPGS^®^	Piperine:HP β CD: TPGS^®^	Piperine:HP β CD: TPGS^®^
1:1	1:1	1:1	1:1:0.05	1:1:0.05	1:1:0.05

**Table 2:** In vitro antimicrobial assay of piperine and its complex against various strains.

Compounds	Zone of Inhibition (mm) *			
	*Staphylococcus aureus* (+)	*Bacillus Subtilis* (+)	*Escherichia coli* (−)	*Enterococcus faecalis* (−)
**Bacterial load (CFU/mL)**	4.5 × 10^−4^	3.0 × 10^−4^	5.8 × 10^−4^	5.0 × 10^−4^
**Pure PIP**	12.4 (0.48)	8.8 (0.78)	11.3 (0.69)	7.2 (0.56)
**PIP-TIC (MI)**	15.7 (0.73)	13.8 (0.46) **	16.4 (0.36) **	11.5 (0.53) **
**PIP-TPM**	13.8 (0.61)	9.3 (0.45)	13.9 (0.35)	8.3 (0.44)
**Distilled water**	0	0	0	0

* Note: Values in the bracket are standard deviation (*n* = 3; mean ± sd); ** (*p* ˂ 0.05) significant difference observed as per ANOVA and Tukey’s multiple comparison tests. PIP, piperine; TIC, ternary inclusion complex; TPM, ternary physical mixture; MI, microwave irradiation; CFU, Colony forming unit.
